# Levodopa-Induced Dyskinesia in Parkinson’s Disease: Pathogenesis and Emerging Treatment Strategies

**DOI:** 10.3390/cells11233736

**Published:** 2022-11-23

**Authors:** Destany K. Kwon, Mohit Kwatra, Jing Wang, Han Seok Ko

**Affiliations:** 1 Neuroregeneration and Stem Cell Programs, Institute for Cell Engineering, The Johns Hopkins University School of Medicine, Baltimore, MD 21205, USA; 2 Department of Neurology, The Johns Hopkins University School of Medicine, Baltimore, MD 21205, USA

**Keywords:** dopamine, levodopa-induced dyskinesia, Parkinson’s disease, neurobiology of disease, treatment

## Abstract

The most commonly used treatment for Parkinson’s disease (PD) is levodopa, prescribed in conjunction with carbidopa. Virtually all patients with PD undergo dopamine replacement therapy using levodopa during the course of the disease’s progression. However, despite the fact that levodopa is the “gold standard” in PD treatments and has the ability to significantly alleviate PD symptoms, it comes with side effects in advanced PD. Levodopa replacement therapy remains the current clinical treatment of choice for Parkinson’s patients, but approximately 80% of the treated PD patients develop levodopa-induced dyskinesia (LID) in the advanced stages of the disease. A better understanding of the pathological mechanisms of LID and possible means of improvement would significantly improve the outcome of PD patients, reduce the complexity of medication use, and lower adverse effects, thus, improving the quality of life of patients and prolonging their life cycle. This review assesses the recent advancements in understanding the underlying mechanisms of LID and the therapeutic management options available after the emergence of LID in patients. We summarized the pathogenesis and the new treatments for LID-related PD and concluded that targeting pathways other than the dopaminergic pathway to treat LID has become a new possibility, and, currently, amantadine, drugs targeting 5-hydroxytryptamine receptors, and surgery for PD can target the Parkinson’s symptoms caused by LID.

## 1. Introduction

**Figure 1:**
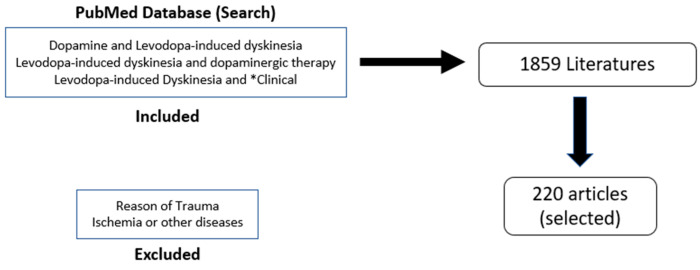
Flow Chart for articles selection and literature search.

Parkinson’s disease (PD) is the second most common neurodegenerative disorder, characterized by progressive motor symptoms, including tremors, bradykinesia, rigidity, and impaired posture [1]. These motor symptoms result from the degeneration of the dopaminergic nigrostriatal pathway, which is the key function of the basal ganglia and responsible for bodily movements [2]. The most effective treatment for PD is l-3,4-dihydroxyphenylalanine (levodopa), which replenishes the dopamine levels in PD. Levodopa is very efficient during the first few years of administration, as in the earlier stages of PD, there are spared dopamine (DA) neurons that are able to store the exogenous dopamine and regulate its release and maintain normal physiological DA receptor stimulation within the striatum [3]. However, as PD progresses, the common motor symptoms of PD are reversed to levodopa-induced dyskinesia (LID), with the levodopa causing involuntary movements [4]. Around 80% of PD patients develop LID, with 30% developing it after just 3 years of levodopa treatments [5,6]. Dyskinesias can become treatment-limiting as increasing the levodopa dose as the disease progresses can exacerbate involuntary movements and decrease the quality of life of patients. As levodopa continues to be used as a treatment, LID becomes progressively worse until it becomes treatment-limiting, as increasing the levodopa dose with the disease progression can exacerbate involuntary movements and decrease the quality of life of patients. LID is rated on several different scales, including the Unified Parkinson’s Disease Rating Scale (UPDRS), the Rush Dyskinesia Scale (RDRS), the Abnormal Involuntary Movement Scale (AIMS), the Parkinson’s Disease Questionnaire-39 (PDQ-39), the Parkinson’s Disease Quality of Life Scale (PDQUALIF), and the Home Diary. The overall goal of the study is to look for the possible anticipated mechanisms involved in LID and the currently available therapeutics that are either studied preclinically or in clinical trials to alleviate the conditions in patients suffering from dyskinesia due to the levodopa medication. This idea of review accomplished with search of literature from PubMed using the keywords “dopamine AND levodopa-induced dyskinesia”, “levodopa-induced dyskinesia AND dopaminergic therapy”, and “levodopa-induced dyskinesia AND *clinical”. The reasons for trauma, ischemia, or other diseases were excluded. Out of the 1859 results, the most relevant 220 were selected for the review (Figure 1).

## 2. Epidemiology and Risk Factors

Among the various neurological disorders, PD is the leading global source of disability in terms of prevalence, disability, and deaths between 1990–2016 [7,8]. The number of people affected by PD globally has more than doubled due to aging populations, increasing life expectancy, lifestyle changes, and, possibly, environmental factors related to the internationally growing industrialization [7,8].

LID manifests in patients as Parkinson’s disease progresses, with dyskinesias becoming more prevalent as the disease reaches its more advanced levels. Previously, LID was thought to be caused by the levodopa administration because the motor fluctuations usually began to set in after 3–5 years of the levodopa intake [9]. Increasing the levodopa dose in patients would also exacerbate abnormal movements, suggesting that levodopa is the direct cause of LID [10]. However, this view has been overturned by recent studies that provide evidence that dyskinesias are more closely related to disease progression than the duration of the levodopa intake [11]. A 2010 study shows that patients in stages 1 or 1.5 did not have motor complications, while 60% of the patients in stages 4 or 5 had LID [12]. The study supports the verdict that the prevalence of LID increases with the disease. However, levodopa may affect how LID manifests in a variety of ways, including the type of formulations administered and the mode of action.

Another main risk factor of LID is the age of onset of PD. Younger patients tend to be at greater risk of developing LID [9,13]. After five years of levodopa administrations, 70% of the patients who developed PD at younger ages, between 40 and 49 years old, developed LID compared to 42% of the patients who developed PD between the ages of 50 and 59 [12]. Women are also more susceptible to LID compared to men [14,15]. Other risk factors include low body weight and native ethnicity of the North American geographic region [14].

## 3. Overview of LID in PD

The three most common types of dyskinesia are peak-dose dyskinesia, diphasic dyskinesia, and early-morning dystonia [16]. These types of dyskinesias are also known as “improvement-dyskinesia-improvement” (IDI) dyskinesia, “dyskinesia-improvement-dyskinesia” (DID) dyskinesia, and off-period dyskinesia, respectively. In general, levodopa-induced dyskinesia (LID) usually starts on the side that is initially affected by PD. These different types of LID are characterized by several different types of abnormal movements, including chorea, ballism, dystonia, and myoclonus [9]. Chorea and dystonia are usually found on the face, mouth, trunk, and limbs, while the others are more typically found on the hands and feet [17]. Chorea is defined as abnormal, involuntary movements characterized by a brief, irregular contraction that is not repetitive. Ballism, or ballismus, is a repetitive, involuntary movement of the limbs that varies consistently. Dystonia involves involuntary muscle contractions that are characterized by slow and repetitive movements or abnormal postures. These movements can affect several different muscles or muscle groups throughout the body. Myoclonus is the sudden twitching or jerking of a muscle or a group of muscles throughout the body [18].

Peak-dose, or IDI, dyskinesia is the foremost form of LID, with 75–80% of patients experiencing this kind of dyskinesia [16]. It occurs during the peak “on” time when the plasma levels of levodopa are the highest, and it was thought to be the only type of LID before the plasma levels of diphasic dyskinesia and off-period dyskinesia were observed [9]. Peak-dose dyskinesia is characterized mainly by choreiform movements, but can involve a variety of movement abnormalities, such as dystonia, myoclonus, or ballism, in the limbs, trunk, or orofacial muscles [19]. Diphasic, or DID, dyskinesia occurs when the plasma concentrations of levodopa are rising and falling [16]. In other words, dyskinesia sets in when levodopa changes from “on” to “off” or “off” to “on”, meaning that whenever levodopa starts supplying dopamine to the brain and when levodopa begins to wear off. This form of dyskinesia is characterized by dystonic or ballistic movements [20]. Early-morning dystonia, or off-period dyskinesia, is usually observed when the plasma levels of levodopa are the lowest [9]. Patients are usually not taking medication during the night, causing the plasma levels of levodopa to be at their lowest when the patients awake. Early-morning dystonia is often characterized by a foot inversion or downward curling of the toes ipsilateral to the side that was initially affected by PD [9,16]. Similar symptoms can be present in early-onset PD patients and are usually differentiated from LID by physicians [21]. The different types of dyskinesia are outlined in Table 1.

## 4. Causes and Mechanisms

When the brain is functioning normally, DA stimulates the direct pathway through D1 receptors and inhibits the indirect pathway via D2 receptors [22]. In PD patients, there is an imbalance between the direct and indirect pathways, with an overactivity of the GPi, which inhibits the motor thalamus, thus, limiting activity in the corresponding motor area [3,23]. At this point, dopamine transporters take up the exogenous dopamine from the levodopa and store it in presynaptic vesicles, so the dopamine levels in these individuals are relatively stable in the synaptic cleft while receiving the levodopa treatment [24]. This can be observed as patients often receive a stable response from the levodopa in the earlier stages of PD. However, as the disease enters a more advanced stage, the severe loss of dopaminergic terminals in the dorsal putamen and the loss of dopamine transporters create an incapacity to store dopamine, releasing more dopamine from the levodopa dose at once [25]. The result is an increased concentration of dopamine in the synaptic cleft when levodopa is administered, causing dopamine receptors in the putamen to be overstimulated, resulting in the most common form of LID, peak-dose dyskinesia [26]. When activated, the D1 receptors lead to the induction of several immediate early genes, including components of the direct pathway. PD patients who have received levodopa treatments have higher D1 receptor numbers in the cytoplasm than healthy people [27], a result of the pulsatile stimulation of the dopamine receptors, which provokes the internalization of the D1 receptors [28]. The levodopa plasma half-life is very short, resulting in marked plasma drug concentration fluctuations, which are matched as the disease progresses to swings in the therapeutic response (the “wearing-off” phenomena). In the more advanced disease stages, the “wearing-off” phenomenon can also be associated with the “negative”, both parkinsonism-exacerbating and dyskinetic, effects of levodopa at low, subtherapeutic plasma concentrations. Dyskinesias may also be related to high levodopa and excessive plasma concentrations. Thus, either low or high doses of levodopa can cause dyskinesia in the late stages, so just improving dopamine instead of the treatment is quite ineffective, and it is important to look for other possibilities [29]. As the levodopa therapy continues, this signaling cascade continues to be enhanced, creating oversensitive D1 receptor signaling in the direct pathway [30]. Most patients with LID are generally experiencing an overactivity of the direct pathway [28]. However, the overstimulation of the D1 receptors and the overactivity of the direct pathway cannot be the only explanation for all forms of LID, as there are dyskinesias that occur when dopamine concentrations are low (off-period dystonia) or when they are rising or falling (diphasic dyskinesia).

The proportions of striatal dopamine receptors change when a patient receives levodopa treatment. A study showed that an increased density of D1 receptors was found in the striatum of LID primates, while no consistent changes were observed for the D2 receptor levels [31]. This implicates the involvement of D1 receptors in dyskinesia, while D2 receptors may be less significant in its pathology. The specific role of D1 agonists in LID is supported by the fact that D1 antagonists may lower the symptoms of LID in parkinsonian patients (Fici et al., 1997). There is also an increase in striatal D3 expression [31,32], and D3 antagonists are able to suppress dyskinesia [32]. These findings point to the conclusion that changes in the striatal dopamine receptor levels are related to dyskinesia induction. There are several treatments for LID that target the dopamine receptors and may reduce the severity of LID.

In PD research, there is a general consensus that pulsatile stimulation of the dopamine receptors is the main mechanism involved in the occurrence of LID. In non-parkinsonian brains, the striatal dopamine receptor activation is continuous, and when levodopa is administered to PD patients, there is a pulsatile stimulation that alters the basal ganglia output [33,34,35]. The pulsatile stimulation results from the short half-life of levodopa, which increases as PD progresses [35,36]. In the early stages of PD, the remaining dopaminergic neurons store the excess exogenous dopamine from the levodopa doses, decreasing the variation of the dopamine levels in the brain [37]. The short half-life of levodopa does not have an impact on the way it works in the body during the early stages of PD because of the storage of excess dopamine in the nigrostriatal dopaminergic terminals. However, as the disease progresses and the dopaminergic neurons continue to be lost, the dopamine is no longer being stored and released properly, so the levels of dopamine in the brain correspond to the plasma levodopa concentrations [38]. While a fluctuating dopamine release is necessary for the development of LID, it is not sufficient on its own [24]; other pathways are factors in the establishment of LID.

Normally, levodopa is decarboxylated to dopamine in the nigrostriatal dopaminergic terminals. The converted dopamine is transported to synaptic vesicles by dopamine transporters, where it is stored [39]. The release of this dopamine into the synaptic cleft is regulated by D2 autoreceptors and dopamine transporters [40]. However, exogenous levodopa may be metabolized in other terminals that express the enzyme aromatic L-amino acid decarboxylase (AADC), namely serotonergic and noradrenergic terminals [24].

**Figure 2:**
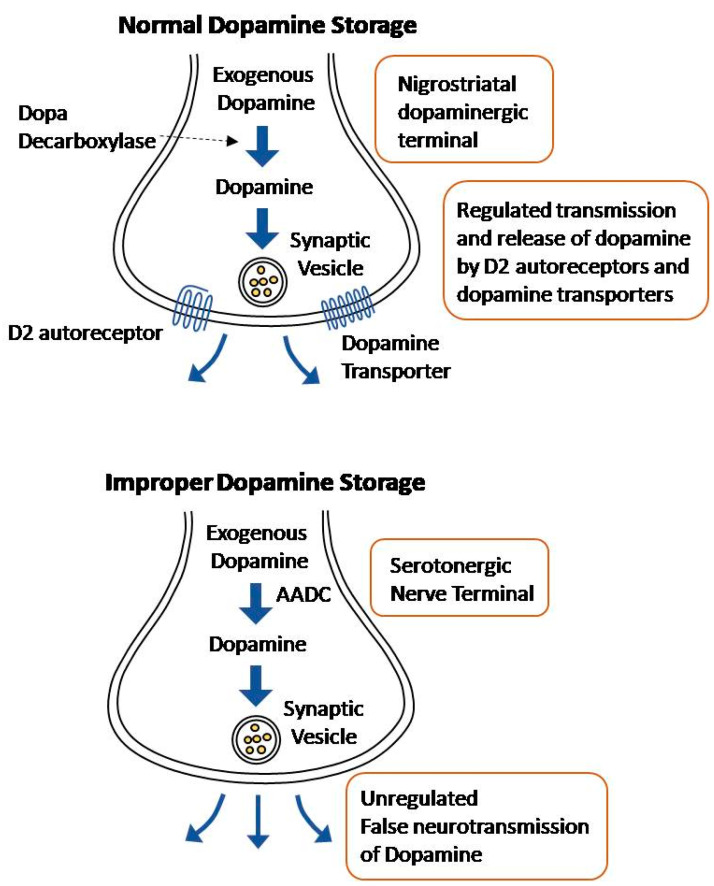
Illustration of Normal dopamine storage Vs improper dopamine storage.

While the serotonergic terminals are able to convert levodopa to dopamine and, thereby, release it, the neurons are unable to regulate the dopamine release due to the lack of D2 autoreceptors and dopamine transporters [41]. Due to the serotonergic neurons’ lack of regulators, dopamine release occurs in an aberrant and sporadic manner (Figure 2). As PD progresses and the dopaminergic terminals continue to die, most of the striatal dopamine is incorrectly released from the serotonergic nerve terminals [39]. This effect is exacerbated by the fact that with disease progression, there is an excessive striatal innervation of serotonergic neurons and a higher serotonin to dopamine striatal terminal ratio [42,43]. The hypothesis that uncontrolled dopamine is released from the serotonergic neurons is supported by the fact that lysis of the serotonin raphe projections blocks LID [44]. Noradrenergic terminals have a similar effect; they express the AADC enzyme, allowing the conversion of levodopa into dopamine, and contribute to the extreme fluctuation in dopamine levels in the striatum [45]. Recently, a new approach of non-dopaminergic systems is prominent in LID management due to its potential methods to appease motor fluctuations without compromising the antiparkinsonian effect of levodopa [28]. Blocking the uptake of serotonin has shown promising results for antidyskinetic action [46].

There are many findings that support the hypothesis that glutamate transmission is significant in the development of LID. There is an increase in spontaneous glutamate release after dopamine denervation, and the level of glutamate released increases after levodopa administration [47]. As a result, patients with dyskinesia have a higher uptake of glutamate in the caudate, putamen, and precentral gyrus [48]. The glutamate neurotransmitters may stimulate neurons through ionotropic glutamate receptors (iGluRs) and metabotropic receptors (mGluRs). Among the iGluRs, the NMDA (N-methyl-D-aspartate) and AMPA (α-amino-3-hydroxy-5-methyl-4-isoxazole propionic acid) receptors are highly involved in the pathology of LID. Both receptors are localized in the striatum of PD patients with LID [49]. There are also abnormalities in the phosphorylation status and subcellular localization of NMDA receptors [50]. The NMDA receptors NR1, NR2A, and NR2B are all hyperphosphorylated and have been found to have an association with the development of LIDs [51]. This conclusion is supported by the fact that amantadine (an NMDA receptor antagonist) is currently the most effective in treating LID without compromising the antiparkinsonian effects of levodopa. Although less effective, AMPA antagonists are also able to alleviate LID, which suggests another conclusion that elevated glutamate levels, in general, play a part in LID development [52].

The loss of nigrostriatal dopaminergic neurons results in several changes, such as the loss of exogenous storage capacity and the expression of at least 50 different genes, which are affected by the chronic loss of dopaminergic input in the striatum [53]. New proteins that are expressed as a result of this alteration may be involved in inducing LID or contributing to motor fluctuations. Plastic changes that occur as a result of dopaminergic death also play a role in how the patient adapts to LID [54].

Some patients exhibit severe dyskinesia in the early stages of the levodopa treatments, while others develop LID much later, despite being on higher doses for a longer period. Neural plasticity could explain this phenomenon if patients adapt to the loss of nigrostriatal dopaminergic neurons differently [16]. One theory suggests that levodopa may cause a stimulatory priming effect in PD patients [55]. The chronic intake of levodopa creates plastic alterations in the postsynaptic region, including a significant rise in extracellular dopamine and defective monoamine oxidase-mediated dopamine breakdown [56].

Several other pathways, such as the PKA/DARPP-32, the ERK, and the mTORC1 signaling pathways, all of which can be triggered by nigrostriatal spinal projection neurons, are interrelated and altered in LIDs [41]. Phosphodiesterase 10 (PDE10), which regulates the PKA/DARPP-32 and cAMP signaling cascades, is involved in the regulation of striatal output and neuronal survival, which may play a role in the induction of LID expression, as PD patients with LID have lower PDE10A levels in their caudate and putamen nuclei [57].

Areas of the basal ganglia that include the substantia nigra, the striatum, the internal or external globus pallidus (GPi and GPe), and the subthalamic nucleus (STN) are involved in the pathology of PD and the onset of LID [41]; in particular, with a focused role of the STN and GPi in the expression of LID. There is a significant boost in the terminal activity of the STN, although this is only seen in dystonic groups and remains normal in patients with choreic movements [28]. On the other hand, there is an underactivity of the GPi and substantia output and a lower cytochrome oxidase messenger RNA expression in the GPi of animals administered with levodopa [28,58]. These findings may lead to the conclusion that LID generally involves underactivity of the basal ganglia output, except for the elevated STN terminal activity with dystonic groups. Furthermore, STN may not be as important for the induction of LID [28] as STN stimulation in DBS does not decrease LID directly but indirectly by allowing a decrease in medication. The stimulation of the GPi, on the other hand, is more effective in reducing LID without improving much in parkinsonian patients [59].

Major inputs into the basal ganglia that may be involved in the occurrence of LID are the dopaminergic nigrostriatal pathway, the glutamatergic corticostriatal pathway, the glutamatergic thalamostriatal pathway, the glutamatergic and acetylcholinergic tracts from the pedunculopontine nucleus, serotonergic inputs from the raphe nuclei, and noradrenergic innervation from the locus coeruleus [2]. Other pathways that could affect the occurrence of LID include glutamatergic, gamma-aminobutyric acid (GABA)-ergic, serotonergic, histaminergic, adenosine, and cannabinoid receptors [60].

**Figure 3:**
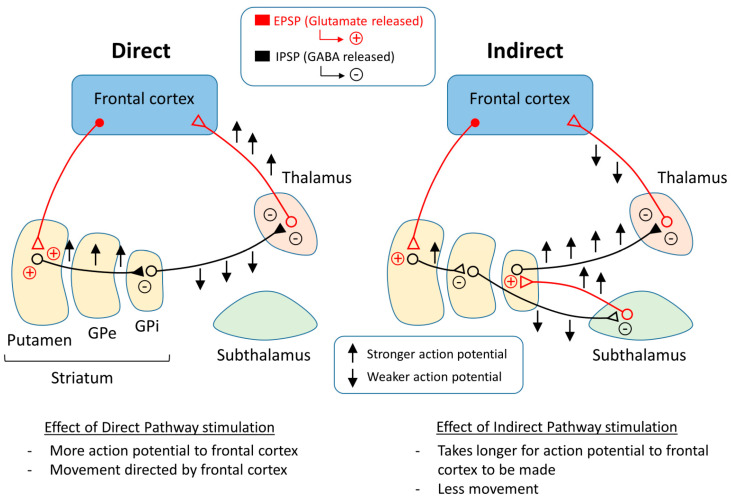
Basal ganglia circuitry regulating the direct and indirect pathways.

**Figure 4:**
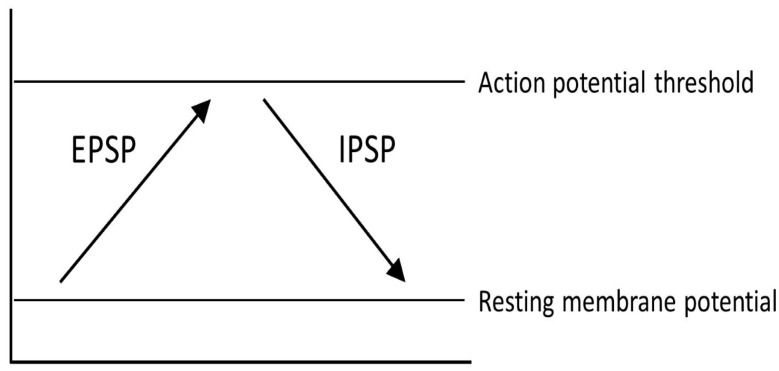
Different physical reactions as a result of excitatory postsynaptic potentials (EPSP) and inhibitory postsynaptic potentials (IPSP).

One representation of PD that tries to explain the changes that occur within the pathways of the brain is the classical model of PD (Figure 3). There are two main pathways described in this model: the direct pathway and the indirect pathway, which follow the effect of dopamine on motor output. The two main neurotransmitters used when explaining the classical model are glutamate and gamma-aminobutyric acid (GABA). Glutamate is excitatory and initiates an excitatory postsynaptic potential (EPSP) when received by receptors, while GABA is inhibitory and initiates an inhibitory postsynaptic potential (IPSP) (Figure 4).

The direct pathway moves from the cortex down the cortical striatal pathway to the putamen and releases glutamate, stimulating an EPSP in the neurons of the putamen and sending a signal to the GPi by releasing GABA. From the GPi, the neurons stretch to the thalamus and release GABA, where action potentials are sent to the cortex as motor output. Thus, if the direct pathway is stimulated, there will be a stronger stimulus received by the frontal cortex, causing muscles to contract or move in some way.

The indirect pathway has a different route to the frontal cortex. In the beginning, the pathway is the same as the direct pathway. The neurons stretching from the cortex to the putamen release glutamate in the putamen and an EPSP is formed. After this point, the pathway changes. Instead of the neurons reaching from the putamen to the GPi, the neurons reach the GPe, where GABA is released. From the GPe, the neurons stretch to the subthalamus, where GABA is released again. The neurons continue toward the GPi and release glutamate. After this point, the pathway is the same as the direct pathway, or from the GPi to the thalamus and then to the motor cortex. In the end, if the indirect pathway is stimulated, there will be a weaker signal to the frontal cortex, which may cause muscles to relax.

To explain Parkinson’s disease, the classical model proposes that the direct and indirect pathways are responsible for controlling the movement of different muscle groups. For example, in the case of biceps and triceps, flexing an arm would require the contraction of the bicep and the relaxation of the tricep. The classical model suggests that the bicep is controlled by the direct pathway and the tricep is controlled by the indirect pathway. As the dopaminergic input is lost, the direct pathway is inhibited, which reduces the voluntary contraction output from the thalamus [33], and the indirect pathway is more active, which also reduces the glutamatergic output from the thalamus to the motor cortex [2]. The indirect pathway activity may cause unwanted relaxation or contraction based on whether it is stimulated. The direct and indirect pathways of the classical model may be able to explain the motor symptoms of PD. Tremors may be caused by alternating contractions, and constant contractions can cause rigidity.

As for the classical model in the context of levodopa treatments, the motor function returns to normal to some extent. Using the classical model to explain this phenomenon, this is caused by less activity of the indirect pathway and more activity of the direct pathway [61]. In the case of LID, the classical model suggests that there is overactivity of the direct pathway, which causes involuntary contractions at random because of the unstable dopaminergic stimulation from the levodopa.

Despite the explanations that the classical model provides for PD, there are several criticisms of the model. New data show that the classical model is most likely not accurate because axons from the striatum send signals to virtually every part of the basal ganglia [62]. The connections described in the classical model are certainly not the only pathways that exist in the system, and there are more inputs and outputs than those accounted for in the model. This complicates the calculation of the inputs and their effect on the outputs, which must be considered when trying to explain the mechanisms of both PD and LID.

## 5. Dopaminergic Management and Treatment

**Figure 5:**
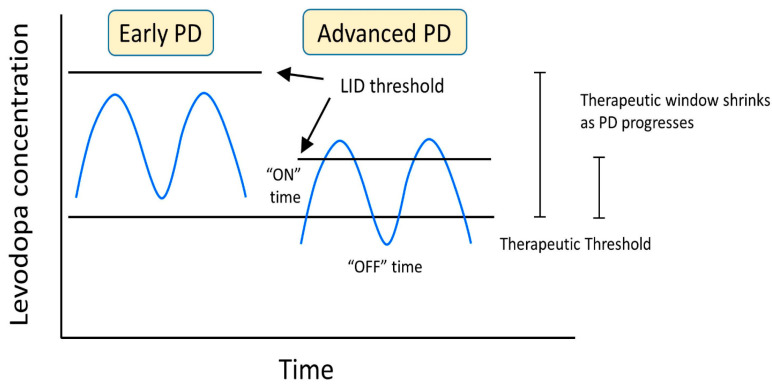
LID developmental changes as a result of levodopa concentration and progression of PD.

When determining the best route of treatment for LID, several factors must be considered, including the time of dyskinesia, the type of dyskinesia, the current medication for PD, the duration of the disease, the stage of Parkinson’s, and the patient’s quality of life. The goal of managing LID is to maximize the patient’s quality of life by reducing the off time of the levodopa and maximizing the time by staying above the anti-parkinsonian threshold amount of the levodopa but under the LID threshold. Maintaining this window of levodopa and motor function can be accomplished by minimizing the fluctuations in the concentration of levodopa and dopaminergic stimulation in the basal ganglia (Figure 5).

The different types of dyskinesia are managed in different ways. Peak-dose dyskinesia, the most common type of LID, is often treated by administering smaller and more frequent doses of levodopa [9], which effectively spreads out the levodopa dosage and decreases fluctuations in the plasma levels of levodopa in the patient. Since the levodopa is not administered all at once, the excess exogenous levodopa is not flooding the brain and causing involuntary movements, but it is still enough to alleviate the parkinsonism. Another popular alternative is switching from controlled-release or long-acting formulations to immediate-acting formulations of levodopa [9]. Immediate-acting formulations may allow for more control when levodopa stimulates the brain and allow doctors to implement the method of prescribing more frequent and smaller doses. If the patient is already on MAO-B or COMT inhibitors, it may be helpful to discontinue the prescription [63].

Treating diphasic dyskinesia can be more complicated because there are two periods when dyskinesia occurs: when levodopa levels are rising and when they are falling. One approach is to decrease the dosage of levodopa and increase the dosage of dopamine agonists [9]. Dopamine agonists have a longer half-life than levodopa, so there are fewer fluctuations when they are used, allowing for more stable dopaminergic stimulation than levodopa and preventing the levodopa levels from changing drastically. Levodopa–carbidopa intestinal gel (LCIG) has also been proven to improve diphasic dyskinesia [64]. LCIG is a gel formulation that is administered through a percutaneous pump with a jejunal extension tube and has been shown to significantly reduce dyskinesia while still providing benefits for the motor and non-motor symptoms of PD, along with an improvement in QoL [65]. A finer titration of levodopa can be administered by using the LCIG pump, which lessens the oscillation of the levodopa levels. However, there may be complications with this surgery, including peristomal complications, problems flushing the tube, accidental removal of the tube, tube occlusion, and weight loss [66].

Off-period dyskinesia, or early-morning dystonia, is less common than the other two types of dyskinesia, but it can be relatively easy to treat. Adding a long-acting formulation at bedtime or adding COMT or MAO-B inhibitors may improve dystonia in the morning [67]. Both would extend the effects of the levodopa by either releasing it later in the night or slowing down the conversion of levodopa into dopamine, allowing the patients to still take the medication in the morning. Another solution is to take the medication right before bed and then again after waking up to minimize the off time. Long-acting dopamine agonists can also decrease the off-period because they have a longer half-life than levodopa [67].

As mentioned previously, dopamine agonists are able to improve several different types of LID (Table 2). Patients treated with ropinirole showed that the risk of LID was significantly lower than that of levodopa-treated patients [68]. Despite the improvements in LID, many doctors are moving away from using dopamine agonists due to their side effects. Ergot dopamine agonists come with a high risk of cardiac valvular, pulmonary, or peritoneal fibrotic disorders [69]. One of the most concerning side effects are compulsive behaviors or impulse control disorders, especially in younger PD patients. The behaviors include addiction to gambling, hypersexuality, compulsive shopping, compulsive eating, punding, and compulsive medication use [70]. These compulsive behaviors cause a significant decrease in QoL and can cause harmful behaviors towards the patients themselves and to others around them or a great loss of money. Screened patients indulge in these compulsive behaviors in correlation with instant gratification, lower cognitive use, and repetitive action [70]. Other side effects of dopamine agonists include psychotic symptoms, such as hallucinations, illusions, and delusions.

In addition to dopamine agonists, there are newer formulations of levodopa that may eliminate LID or reduce its severity, including XP21279, CVT-301, L-DOPA/Benserazide microspheres (LBM), chitosan-coated nanoliposomes (CCN), ND0612, and the Accordion Pill (Table 3). As mentioned previously, COMT and MAO-B inhibitors have both shown a degree of success in the management of LID (Table 2). COMT inhibitors slow down the breakdown of levodopa and dopamine, which can allow for less dramatic fluctuations in the endogenous dopamine levels. Examples include entacapone and tolcapone, although tolcapone comes with possible severe side effects, such as liver toxicity [41]. MAO-B inhibitors work in a similar way and slow down the breakdown of levodopa into dopamine, but they can also be used in monotherapy, while COMT inhibitors are only used in conjunction with levodopa [98]. One example is safinamide, a glutamate release inhibitor and an MAO-B inhibitor [9], which has been shown to increase the “on” time and improve the UPDRS III score [99,100]. A study confirmed that safinamide could be a good option to add to dopaminergic treatments in PD patients, while other agents may contribute to LID [99].

## 6. Non-Dopaminergic Management and Treatment

Amantadine, an NMDA receptor antagonist [2,61], is widely recognized as the most effective drug for LID (Vijayakumar & Jankovic, 2016). A double-blind, placebo-controlled, crossover study in 2000 found a 24% reduction in the total dyskinesia score following amantadine intake without reducing the benefits of levodopa on parkinsonism [113]. Unfortunately, experiences with rebounds in dyskinesia after long-term amantadine use have been seen [114], but the AMANDYSK trial, a 3-month, multicentered, randomized, double-blind, placebo-controlled, parallel-group, wash-out study with 57 patients, found that “on” time with troublesome dyskinesia worsened on the UPDRS scale following the termination of the amantadine medication. The results also observed that amantadine continues to be effective in treating dyskinesia for over a year without wearing off. Because amantadine is an NMDA receptor antagonist, its effectiveness suggests that the overactivity of the glutamatergic input in the basal ganglia is associated with dyskinesia. Other glutamatergic targets have emerged since. For example, rabphilin 3A (Rph3A), a binding partner of the NMDA receptors containing the GluN2A subunit, has been linked to the aberrant synaptic localization of GluN2A-expressing NMDA receptors, which are characterized in LID [115]. This presents a novel therapeutic target for LID treatments. Another target related to NMDA function is one of its key regulators, the kinase Fyn. Fyn mRNA was reduced with RNA interference therapy in adult mouse models of PD, resulting in the prevention of LID onsets [99]. More than a possible option for management, this target creates an opportunity for intervention before the onset of LID.

A promising target is the serotonergic system. As mentioned, serotonergic neurons are able to convert exogenous levodopa into dopamine and release it, but they are unable to regulate the release properly, causing excessive stimulation of the striatal DA receptors [3]. There are several types of 5-HT receptor subtypes that are targeted, including 5-HT_1A_R (dorsal raphe nucleus and striatum), 5-HT_1B_R (striatopallidal pathways), and 5-HT_2A_R (substantia nigra pars reticulata and globus pallidus internus) (Table 4). Eltoprazine, a 5-HT_1A/B_ agonist, is highly effective in blocking LID in 6-OHDA-lesioned rats and in MPTP-treated macaques [116]. A double-blind, randomized, placebo-controlled, and dose-finding phase I/IIa study showed that 5 mg significantly reduced LID on the Clinical Dyskinesia Rating Scale and the Rush Dyskinesia Rating Scale. However, it also causes a slight reduction in the efficacy of levodopa, which is a concern for clinical applications [3]. The management of LID through serotonergic manipulation will only be used if it is more effective than amantadine, which is widely considered the best drug for LID and does not cause any reduction in the parkinsonian benefits of levodopa.

Adrenergic receptor antagonists have shown potential in LID management (Table 4) through similar mechanisms. The α2 adrenergic receptor antagonists yohimbine, rauwolscine, and idazoxan have resulted in a reduction in LID in MPTP-lesioned primates [117,118], and fipamezole (JP-1730), another potent antagonist, has been able to reduce LID without reducing the anti-parkinsonian effect of levodopa. Fipamezole is promising because it not only does not reduce the action of levodopa but also increases its duration of efficacy and reduces dyskinesia with great magnitude, allowing animals that previously had severe levels of involuntary movement to perform coordinated motor actions [119]. Adrenergic receptor antagonists have shown promise beyond animal models of PD and LID; in a double-blind, randomized, placebo-controlled, dose-escalating 28-day study with 189 PD patients, fipamezole was able to treat LID without taking away the levodopa’s antiparkinsonian effects at 90 mg three times a day. However, their successful results are mixed. In other studies of fipamezole and idazoxan, there was some improvement in dyskinesia with US patients but not with Indian patients [120].

**Table 4:** Non-Dopaminergic Medications.

Targeted System	Mechanism of Action	Side Effects	Name of Drug	Route of Administration	References
Adenosine	Adenosine A_2A_ receptor antagonist	Falls in systolic and diastolic blood pressure, nausea, dizziness, insomnia, stiffness, vomiting, headache, hallucinations	ST1535	Oral	[121,122]
			Istradefylline (KW-6002)	Oral	[123]
			Preladenant (SCH-420814)	Oral	[121,124]
			Tozadenant (SYN115)	Oral	[121,124]
			Vipadenant (V2006)	Oral	[121,124]
			Ciforadenant (CPI-444, V81444)	Oral	[121,124]
			PBF-509	Oral	[121,124]
			ST4206	Oral	[121,124]
	Nonspecific adenosine receptor antagonist	N/A	Caffeine	Oral	[125]
Adrenergic	Alpha-2 adrenergic receptor antagonist	Nausea, vomiting, dysgeusia, headache, oral hypoesthesia, flushing, and increases in systolic and diastolic blood pressure; potentially severe adverse effects affecting the cardiovascular system	Idazoxan	Oral	[20]
			Yohimbine	Oral	[126]
			Fipamezole (JP-1730)	Oral	[127]
	Beta-2 adrenergic receptor antagonist	Risk of bronchospasm, masked symptoms of hypoglycemia, bradycardia, hypotension, dizziness, lowered heart rate and blood pressure	Propranolol	Oral	[128]
			Salbutamol	Oral	[129]
Glutamatergic	NMDA receptor antagonist	confusion, worsening of hallucinations, peripheral oedema, skin rash, re-emergence of palpitations, nausea, dry mouth, swelling of feet and constipation	Amantadine & Amantadine extended-release capsules (Gocovri^®^)	Oral	[113,130,131]
		Amnesia, dissociation	Traxoprodil	Oral	[132]
		tiredness, vertigo, increased off time	Memantine	Oral	[133]
		Neurotoxicity, hyperactivity	Dizocilpine (MK-801)	Oral	[134]
		Perceptual distortions, detachment, anxiety, nausea, dissociation, confusion	Ketamine (FDA Approves IND for Ketamine in Parkinson Disease Dyskinesia, 2021)	Intravenous infusion, oral	[135,136]
	GluN2B antagonist	Amnesia, dissociation	Ifenprodil	Oral	[132]
			Traxoprodil (CP-101606)	Oral, intravenous infusion	[132]
	AMPA receptor antagonist	Dry eyes and mouth, hallucinations, worsening dyskinesia, anxiety/depression, breathing problems	Topiramate	Oral	[137]
		Somnolence, dizziness, worsening of dyskinesia	Perampanel	Oral	[138]
		N/A (ongoing clinical trial)	Talampanel (LY-300164)	N/A (ongoing clinical trial)	[132]
			Tezampanel (LY-293558)	N/A (ongoing clinical trial)	[132]
	mGlu4R positive allosteric modulator	N/A (preclinical)	ADX88178	N/A (preclinical)	[132]
			Lu AF21934	N/A (preclinical)	[132]
		on and off phenomenon, dyskinesia, headache	Foliglurax	Oral	[139]
	mGluR5 receptor antagonist	Dizziness, hallucination, fatigue, nasopharyngitis, diarrhea, insomnia	Mavoglurant	Oral	[140]
	mGlu5R negative allosteric modulator	N/A (preclinical)	MPEP [2-Methyl-6-(phenylethynyl)-pyridine]	N/A (preclinical)	[141]
			MTEP (3-[(2-methyl-1,3-thiazol-4-yl)ethynyl]-pyridine)	N/A (preclinical)	[141]
		memory loss, dizziness, hallucinations	Fenobam	Oral	[142]
		Dizziness, nausea	Dipraglurant (ADX48621)	Oral	[141]
	mGlu7R allosteric agonist	N/A (preclinical)	AMN082 (N,N′-dibenzhydrylethane-1,2-diamine)	N/A (preclinical)	[143]
Serotonergic	5-HT_1A_R agonist	Dizziness, increase in “off” time	Buspirone	Oral	[144]
		N/A (ongoing clinical trial)	Befiradol (F-13,640; NLX-112)	N/A (ongoing clinical trial)	[145]
		N/A (preclinical)	F13714	N/A (preclinical)	[146]
	5-HT_1B_R agonist	N/A (preclinical)	CP-94253	N/A (preclinical)	[147]
	5-HT_1A_R/5-HT_1B_R agonist, 5-HT_2C_R antagonist	Fatigue, nausea, dizziness, mild somnolence/sedation	Eltoprazine	Oral	[88]
	5-HT_1A_R agonist, partial D2 agonist	Worsening of parkinsonism, falls, aggravated tremor, somnolence, fatigue, headache, and arthralgia	Sarizotan	Oral	[148]
	Partial 5-HT_1A_R agonist	Sedation, muscle relaxation	Tandospirone	Oral	[149]
	5-HT_2A_R inverse agonist and antagonist	Urinary tract infections, falls	Pimavanserin (ACP-103)	Oral	[150]
	5-HT_2A_R, 5-HT_2C_R antagonist, D1R agonist, D2R, D4R antagonist	Diurnal drowsiness, somnolence, excessive sweating, hypereosinophilia, worsening of parkinsonism	Clozapine	Oral	[151]
	5-HT_2A_R agonist and adrenergic, muscarinic, histaminergic, dopaminergic receptor action	Sedation, dizziness	Quetiapine	Oral	[152]
	5-HT_2A_R antagonist, partial 5-HT_1A_R agonist, partial D2 agonist	headache, insomnia, agitation, anxiety, dyspepsia, nausea, lightheadedness, somnolence, akathisia	Aripiprazole	Intramuscular injection, oral	[153]
	5-HT_2_R agonist/5-HT_3_R antagonist	Hallucinations, confusion	Mirtazapine	Oral	[154]
		Worsening of parkinsonism	Mianserin	Oral	[132]
Histamine	H3 antagonist	N/A (preclinical)	Thioperamide	N/A (preclinical)	[155]
	H3 receptor agonist	N/A (preclinical)	Immepip	N/A (preclinical)	[156,157]
		N/A (preclinical)	Imetit	N/A (preclinical)	[157]
Cholinergic	M1 receptor antagonist	dry mouth, blurred vision, constipation, confusion, hallucination, memory disturbance, urinary retention	Trihexyphenidyl	Oral	[158]
			Benzatropine	Oral	[159]
	M4 positive allosteric moderator	N/A (preclinical)	VU0467154	N/A (preclinical)	[132]
	M4 positive allosteric moderator	N/A (preclinical)	VU0476406	N/A (preclinical)	[132]
	Nicotinic agonist	nausea, vomiting, lung damage, dizziness,	Nicotine	Oral, inhaled	[160,161]
	Non-selective nicotinic receptor antagonist	constipation, blurred vision, dry mouth, orthostatic hypotension	Mecamylamine	Oral	[162]
	ɑ7 nicotinic receptor agonist	N/A (preclinical)	ABT-107	N/A (preclinical)	[163]
		agitation, constipation, diarrhea, fall, and headache	ABT-126	Oral	[163,164]
	ꞵ2 nicotinic receptor agonist	Worsening of parkinsonism	ABT-089	Oral	[163]
			ABT-894	Oral	[163]
Opioid	к agonist, μ antagonist	Sedation (Non-human primate models)	Nalbuphine	Injection (Non-human primate models)	[165]
	Mu-delta opioid receptor agonist	N/A (preclinical)	Lactomorphin (MMP-2200)	N/A (preclinical)	[166]
	δ antagonist	N/A (preclinical)	Naltrindole	N/A (preclinical)	[167]
	Kappa (*к*) opioid receptor agonist	Worsening of parkinsonism	U50,488	N/A (preclinical)	[168]
Sigma-1	σ1 receptor antagonist	N/A (preclinical)	BMY-14802	N/A (preclinical)	[169]
	σ1 receptor agonist	Fatigue, somnolence, dizziness, constipation	Dextromethorphan	Oral	[170]
Nitric Oxide	NO Donor	Worsening of parkinsonism	Molsidomine	Oral	[171]

There is a strong link between adenosine and the function of other neurotransmitter pathways. Adenosine and adenosine A2 receptors are able to regulate dopaminergic, GABAergic, glutamatergic, and cholinergic activities [172]. A2 receptors work especially closely with dopamine receptors. They have a strong relationship with D2 receptors in the striatum because they colocalize with D2 receptors in the striatum [173] and also have a strong link to D3 receptors [174]. This is demonstrated when the adenosine A2 receptors are activated and a reduction in the D2 receptor recognition results, effectively creating an inhibitory effect on the D2 receptor signaling [174]. In the end, this causes overactivity of the indirect pathway by moderating the D2 and D1 dopamine receptors [173]. In addition to the already close relationship between adenosine and dopamine receptors, there are changes in the density of the adenosine receptors in LID pathology. Macaques with LID have been found to have higher A2a receptor mRNA levels in the striatum [175] and have proven to be a point of management for certain drugs. For example, NMDA receptor antagonists that lowered the levels of the A2a receptor mRNA have prevented LID [176]. As for the testing with PD patients afflicted with LID in the monkeys challenged with MPTP, there were mixed results on the efficacy of the adenosine drugs. KW-6002, an A2a antagonist, has shown improvements in the motor symptoms of PD with less dyskinesia in a study of 15 patients [177], but in a double-blind, randomized, multicenter clinical trial with 196 patients, there was an increase in non-troublesome dyskinesia [124,178]. Caffeine, a nonspecific adenosine receptor antagonist, is also able to alleviate some of the LID symptoms [179]. More research is needed to prove the potential of adenosine A2 receptor antagonists.

In addition to the close connection between adenosine and dopamine receptors, histamine receptors H1 and H3 are also located on the striatal dopaminergic terminals in both rats and humans with PD, as shown by how histamine-inhibited dopamine release in superfused mouse striatal slices through presynaptic H3 receptors [180]. The striatum is densely innervated by histaminergic projections, with H2 and H3 receptors highly expressed in the basal ganglia [120]. In addition to their locational correlation, histamine receptors may alter the striatal levels of dopamine, GABA, acetylcholine, and glutamate by acting as heteroreceptors [174]. Like the serotonergic and adrenergic neurons, histaminergic neurons are also able to take up levodopa and convert it to dopamine [181], making it a promising target for managing LID. Indeed, the histamine H3 hetero-receptor agonists have had some success in preventing LID. Immepip, a potent H3 receptor agonist, was able to reduce choreic dyskinesia in MPTP-lesioned non-human primate models of PD with LID [157], although it was less effective in preventing dystonia. The chronic administration of immepip was also able to prevent LID in 6-OHDA-lesioned rats [156]. However, there are limitations to the success of histaminergic drugs. In the same study, LID was only prevented in the rat PD models with continuous administration of immepip; single and acute doses had no LID-preventing ability. Higher doses of the histaminergic agonists without levodopa also worsened the parkinsonian symptoms in MPTP-lesioned marmosets [157]. Like adenosine drugs, more research needs to be performed in order to rely on histamine receptors as targets for LID management.

Acetylcholine targets both muscarinic receptors and nicotinic acetylcholine receptors [182], and both targets demonstrate a possibility for LID management. Early into the research of Parkinson’s disease, there was an observed imbalance of acetylcholine and dopamine that increased as the disease progressed, and it eventually resulted in the overactivity of cholinergic interneurons. Due to this, muscarinic antagonists were one of the first treatments for PD [183] and remain a viable option as an adjunctive medication for the treatment of PD [182] (Table 4). In addition to their parkinsonian benefits, muscarinic antagonists suggested to alleviate LID, especially in younger patients [132]. However, their use is limited due to their side effects, which include confusion, hallucinations, dry mouth, memory disturbance, and urinary retention [184]. Nicotinic receptors, which can be expressed on dopaminergic neurons, indirectly affecting dopamine release, have also been able to reduce LID by 60% in several different PD animal models, including mice, marmosets, and macaques [185]. In addition, nicotinic receptors are localized on the GABAergic, serotonergic, and glutamatergic interneurons [186], meaning that they are able to modulate the release of these neurotransmitters and affect LID and PD in indirect ways as well.

In PD patients with dyskinesia, there are several significant changes in opioid signaling. Both densities of mu and delta binding sites are elevated in dyskinetic patients. Even within non-dyskinetic patients, there is an increase in kappa radioligand binding [187]. The levels of the opioid peptides enkephalin and dynorphin are also increased in the striatum, thalamus, and anterior cingulate cortex of dyskinetic PD patients [188]. Since the aberrant dopaminergic release increases as the disease progresses, the levels of endogenous opioids also rise in parallel. This increased activity of the opioidergic pathways is correlated with LID, leading researchers to target them as a novel form of dyskinesia management. However, up to now, due to their high complexity in mode of action, there have been mixed results for the efficacy of opioid drugs [174]. One promising opioid receptor antagonist is nalbuphine, which works by blocking the overexpression of ΔFosB, prodynorphin, dynorphin A, and cyclin-dependent kinase 5 and the increase in the phosphorylation of DARPP-32, in addition to the antagonism at opioid receptors [165]. It was able to reduce LID by 48% without compromising the efficacy or changing the plasma levels of levodopa in MPTP-treated macaques [165]. There was also no tolerance developed toward the drug. Although nalbuphine is promising, it still has side effects, including nausea, sedation, and dizziness (Table 4). Another problem is that due to their high complexity in the mode of action, there have been mixed results for the efficacy of opioid drugs [174].

Nitric oxide (NO) overproduction may play a role in the occurrence of LID. The onset of dyskinesia is correlated with changes in the levels of NO synthase mRNA [189]; in fact, 6-OHDA-lesioned rats chronically treated with levodopa upregulate striatal NO synthase mRNA [190]. NMDA and AMPA receptor activations also stimulate NO synthesis, linking their production to glutamatergic functions [191]. However, there has also been evidence for the involvement of NO underactivity in the pathology of LID. There is a significant decrease in the expression of neuronal NO synthase-containing neurons and mRNA in PD patients [41]. The underactivity of NO is supported by the fact that molsidomine, an NO donor, administered with levodopa, may decrease the extent of LID, supposedly by increasing the expression of dopamine and lowering the need for exogenous dopamine from the levodopa [192]. The involvement of NO in LID is complex, and more research is needed to understand exactly how it plays a role in PD.

Sigma-1 (σ1) receptors are emerging as a new target for LID (Table 4). Recent findings show that they are downregulated in early-stage PD patients and that stimulation of the σ1 receptors improves LID and may have possible neuroprotective properties for PD patients [193]. Still, they are less thoroughly researched than the other targets of LID management and need more research and testing before they can be used as an effective strategy.

Antiepileptic drugs that have been tested for the treatment of LID include zonisamide, topiramate, and levetiracetam. Zonisamide, which works through the inhibition of glutamate release, MAO-B inhibition, and increases in dopamine synthesis, has had positive results in several studies, and it has been shown to reduce the off-time in PD patients with diphasic dyskinesia [9,194]. Topiramate reduced LID in rats, but it was found to worsen dyskinesia in a randomized, double-blind, crossover trial with 15 patients [137]. Levetiracetam reduced the severity of dyskinesia in MPTP-induced macaques, and several open-label studies have shown a significant reduction in the severity of LID [194].

Clozapine and quetiapine, both of which are antipsychotic drugs, have both been used to treat LID. In several open-label studies, including a double-blind, placebo-controlled trial, the results reported that clozapine is effective in reducing the severity of LID. The proposed mechanisms of clozapine include antagonistic binding to the D2 dopamine receptors and to the serotonin type 2A (5-HT_2A_) receptors [19]. The possible side effects of clozapine include somnolence, sialorrhea, seizures, orthostatic hypotension, myocarditis, agranulocytosis, and asthenia. Quetiapine is a 5-HT_2A_ antagonist that has shown a minimal reduction in dyskinesia, but these results were offset by reported drowsiness and daytime somnolence [195].

## 7. Surgical Options

For patients who receive high benefits from levodopa regarding parkinsonian movements, but experience disabling dyskinesia despite other forms of medical management, deep brain stimulation (DBS) decrease LID while still achieving the same extent of improvement in parkinsonian symptoms. There are two main targets for DBS treatment in PD patients: the subthalamic nucleus (STN) and the globus pallidus (GPi). Both achieve improvements in parkinsonian symptoms and relieve dyskinesia but are hypothesized to work through different mechanisms. DBS that targets the STN has been argued to alleviate LID by allowing a reduction in the levodopa dosage by 50 or 60% [196]. However, a recent evaluation with 133 patients has shown that there is a reaction in dyskinesia without any reduction in medication [197]. One postulated explanation is that DBS stimulation of the STN causes plastic changes in the basal ganglia circuits that regulate the L-dopa response [198]. As for the GPi-targeting DBS, it is widely accepted that the stimulation of the GPi affects LID more directly [197]. The GPi has also been found to produce greater antidyskinetic effects [199].

As mentioned before, the infusion of LCIG, also known as Duodopa, is another option for LID management that has shown significant improvements in dyskinesia. The surgery involves the installation of a jejunal extension tube attached to a percutaneous pump, where the gel formulation of levodopa can be administered. However, there is a relatively high frequency of complications, leading many doctors to use surgery only as a last resort. Another concern is that continuous stimulation may induce tolerance to levodopa instead of sensitization to it [200].

Continuous subcutaneous apomorphine infusion may also be an option for those who cannot afford or are not good candidates for DBS. Apomorphine is non-narcotic morphine that acts as a dopamine agonist at the D1 and D2 receptors [19] with a short half-life and rapid results. Apomorphine can be continuously administered through a subcutaneous catheter attached to a pump. Another alternative is to inject it periodically through a pen. One study with 12 PD patients with on–off fluctuations and disabling dyskinesia showed significant improvements over 6 months [80]. The continuous infusion of apomorphine is less invasive than DBS surgery and may be more cost-effective for those who are unable to afford DBS or LCIG. However, this may not be an appropriate treatment for elderly patients because the doses are often not tolerated, leading to confusion, psychosis, or worsening of the existing dysautonomia [19].

Transcranial magnetic stimulation (TMS) is a non-invasive procedure that stimulates certain cells in the brain using magnetic waves. In a study of low-frequency TMS with 17 PD patients with LID, there was a significant improvement in peak-dose dyskinesia that lasted for up to 24 h [201]. However, due to the brevity of the procedure’s effects, this treatment may not be plausible for the long-term treatment of LID. Previously, stereotactic radiofrequency pallidotomy has shown improvements in the motor fluctuations of PD patients, especially LID [202]. Transcranial magnetic resonance-guided focused ultrasound (TcMRgFUS) is a more non-invasive approach to conducting a pallidotomy. In a 1-year open-label trial with twenty patients with PD and motor fluctuations, the majority of the patients showed significant improvements in the impairment scores of the UDysRS and on the motor component of the UPDRS, supporting the MRgFUS pallidotomy for the treatment of PD patients with motor fluctuations, including LID [203].

## 8. Conclusions

LID is a common side effect that often cannot be avoided after initiating dopamine replacement therapy with levodopa. The three types of dyskinesia, peak-dose dyskinesia, diphasic dyskinesia, and off-period dyskinesia, vary in their treatments, and there are a variety of options for adjusting the levodopa doses or using different levodopa formulations according to each type. Although the exact mechanism of LID is unknown, there is an understanding that several pathways are involved in its pathology, with the foremost being the dopaminergic pathway. Other pathways that may be involved include the adenosinergic, adrenergic, glutamatergic, serotonergic, histaminergic, cholinergic, opioidergic, Sigma-1, and nitric oxide pathways. Various drugs target pathways other than the dopaminergic pathway for the management of LID in order to find an adjunctive treatment that would be able to assuage dyskinesia in PD patients without subtracting from the full antiparkinsonian effects of levodopa. The amantadine remains to be the best option for levodopa management, but drugs targeting serotonergic receptors are garnering much interest, as they have successfully reduced the severity of LID without affecting the function of levodopa. Another increasingly viable option for LID management is surgeries for PD, as they often allow for a decrease in the levodopa dosage, indirectly decreasing the symptoms of LID. However, there are many preclinical drugs that have had much success in animal models of PD with LID without any clinical trials.

Another prevalent issue is that many of the drugs with novel targets have serious side effects that eliminate them as a possibility for LID management. Drugs that are without side effects but cause a reduction in the efficacy of levodopa’s antiparkinsonian function are also limited in clinical use because amantadine already exists as an effective option that does not cause a decrease in levodopa function. In order to be used in practice, new therapeutic approaches to LID will be needed to produce results that reduce LID more effectively than amantadine without impacting the function of levodopa.

These pathways, other than the dopaminergic pathway, have become a new possibility to treat LID, and, currently, amantadine, drugs targeting the 5-hydroxytryptamine receptors, and surgery for PD can target Parkinson’s symptoms caused by LID. Other pathways, including the adenosine, adrenergic, glutamatergic, and histaminergic pathways, have found great success in animal models; however, further clinical trials are needed to validate them.

## Figures and Tables

**Table 1:** Different types of dyskinesia.

Dyskinesia Types	Movement	Body Part	Occurrence
Peak dose (IDI)	Primarily choreiform movement; may also be characterized by dystonia, myoclonus, or ballism	Primarily neck, axial, and proximal limbs	High plasma levels, coincidence with antiparkinsonian benefit
Diphasic (DID)	Repetitive dystonia, ballism	Lower limbs	Plasma levels rise or fall at the start of the levodopa effect and as levodopa wears off
Off-period Dystonia (Early Morning Dystonia)	Dystonia	Usually, one foot	Low plasma levels, “off” period

**Table 2:** Levodopa Adjunctive Dopaminergic Medications.

Mechanism of Action	Name of Drug	Route of Administration	Side Effects	References
Dopamine receptor agonists	Cabergoline (ergot D2 agonist)	Oral	Nausea, postural hypotension, somnolence, sleep disorder, hallucination	[71]
	Dihydroergocryptine (ergot D2 agonist, partial D1 agonist)	Oral	Nausea, vomiting, dry mouth, diarrhea, abdominal pain	[72]
	Bromocriptine (ergot partial D2 agonist)	Oral	Nausea, postural hypotension, hallucination, vomiting, dizziness,	[73]
	Pergolide (ergot D2/D3 agonist)	Oral	Nausea, hallucination	[74]
	Pramipexole (non-ergot)	Oral	nausea, vomiting, postural hypotension, oedema, hallucinations, somnolence, sudden-onset sleep disorder	[75]
	Ropinirole (non-ergot D2 agonist)	Oral	Nausea, hallucination, postural hypotension	[73]
	Rotigotine (non-ergot D3/D2/D1 agonist)	Transdermal patch	nausea, vomiting, somnolence, dizziness and application-site reactions	[76]
	Piribedil (non-ergot D2/D3 agonist)	Oral	nausea, postural hypotension, vomiting, confusion, hallucinations, agitation, dizziness, drowsiness	[77]
	Apomorphine (non-ergot D2 agonist)	Intermittent subcutaneous injection or continuous subcutaneous infusion	Administration site reactions, nausea, vomiting, transient sedation, somnolence, dizziness, confusion, hallucinations	[78,79,80]
	Tavapdon (D1/D5 agonist)	Oral	headache, nausea, and vomiting	[81]
	DETQ (D1 agonist)	N/A (preclinical)	N/A (preclinical)	[82]
	SK609 (D3 agonist)	N/A (preclinical)	N/A (preclinical)	[83]
	D-512 (D2/D3 agonist)	N/A (preclinical)	N/A (preclinical)	[84]
	D-636 (D2/D3 agonist)	N/A (preclinical)	N/A (preclinical)	[85]
	D-536 (D2/D3 agonist)	N/A (preclinical)	N/A (preclinical)	[85]
	D-656 (D2/D3 agonist)	N/A (preclinical)	N/A (preclinical)	[85]
	Pardoprunox (D2/D3 partial agonist, 5-HT_1A_ receptor agonist)	Oral	Nausea, vomiting, dizziness, somnolence, headache, insomnia, hallucination	[86]
D4 antagonist	VU6004461	N/A (preclinical	N/A (preclinical)	[87]
D3 antagonist	IRL790	Oral	Asthenia, dissociation, headache, worsening of parkinsonism	[88]
COMT inhibitor	Entacapone	Oral	Diarrhea, nausea, constipation, abdominal pain	[89,90]
COMT inhibitor	Tolcapone	Oral	dyskinesia, nausea, sleep disorders, dystonia, orthostatic hypotension, diarrhea, dizziness, hallucinations, potential elevated liver transaminase concentrations, possible fulminant hepatic failure	[91]
COMT inhibitor	Opicapone	Oral	Dry mouth, dizziness, nausea, constipation, insomnia, hallucination, fall	[92]
MAO-B inhibitor	Safinamide	Oral	Headache, hypertension, cataract, back pain	[93,94]
MAO-B inhibitor	Selegiline	Oral	insomnia, nausea, benign cardiac arrhythmias, dizziness and headache	[95]
MAO-B inhibitor	Rasagiline	Oral	Infection, headache, musculoskeletal pain	[96,97]

**Table 3:** Levodopa Formulations.

Name of Drug	Mechanism of Action	Route of Administration	Side Effects	References
XP21279	L-DOPA prodrug, activated by carboxylesterase, creates more stable plasma concentration	Oral	Headache, dizziness, anorexia, insomnia, gastroesophageal reflux disease, and somnolence	[101,102]
CVT-301	L-DOPA powder	Inhaled	Dizziness, cough, and nausea	[101,103]
L-DOPA/benserazide microspheres	Inactivity of D1R/Shp-2/ERK1/2 pathway, inhibition of tau protein phosphorylation and PKA signaling	N/A (preclinical)	N/A (preclinical)	[104,105]
Chitosan-coated nanoliposomes	Reduced expression of ERK ½, DARPP-32, and FosB/ΔFosB	N/A (preclinical)	N/A (preclinical)	[106]
ODM-101	LD/CD/ENT formulation	Oral	nausea, dizziness, headache, diarrhea, and insomnia	[107,108]
ND0612	Liquid formulation of LD/CD, continuous administration	Transcutaneous, patch-pump device	Small transient papules at infusion sites, infusion site bruising, and erythema	[101,109]
LCIG	Gel formulation of LD/CD, finer and more continuous titrations of levodopa	Intestinal, injected through PEG-J tube	peristomal complications, problems flushing the tube, accidental removal of the tube, tube occlusion, weight loss, nausea, and hallucinations	[66,110]
Accordion Pill	Immediate-release CD and both immediate and extended-release LD	Oral	Nausea, vomiting, mild somnolence, and fatigue	[111,112]

LD = levodopa, CD = carbidopa, ENT = entacapone.

## Data Availability

Not applicable.

## Abbreviations

LID: Levodopa-induced dyskinesia; PD: Parkinson’s disease; DA: Dopamine; UPDRS: Unified Parkinson’s Disease Rating Scale; AIMS: Abnormal Involuntary Movement Scale, PDQ-39: Parkinson’s Disease Questionnaire-39, PDQUALIF: Parkinson’s Disease Quality of Life Scale; LEVODOPA: L-3,4- dihydroxyphenylalanine; AADC: L-amino acid decarboxylase; iGluR: Ionotropic glutamate receptors; mGluR: Metabotropic receptor; NMDA: N-methyl-D-aspartate; AMPA: α-amino-3-hydroxy-5-methyl-4-isoxazole propionic acid; GPi: Globus pallidus internal; GPe: Globus pallidus external; STN: Subthalamic nucleus; MAOB: Monoamine oxidase B; COMT: Catechol-O-methyltransferase; GABA: Gamma-aminobutyric acid; EPSP: Excitatory postsynaptic potential; IPSP: Inhibitory postsynaptic potential; DBS: Deep brain stimulation.
